# Enhancing Deep Learning-Based Segmentation Accuracy through Intensity Rendering and 3D Point Interpolation Techniques to Mitigate Sensor Variability

**DOI:** 10.3390/s24144475

**Published:** 2024-07-11

**Authors:** Myeong-Jun Kim, Suyeon Kim, Banghyon Lee, Jungha Kim

**Affiliations:** 1 Graduate School of Automotive Engineering, Kookmin University, Seoul 02707, Republic of Korea; 2 Moovita Pte Ltd., Block 44, 535 Clementi Rd., Singapore 599489, Singapore; 3 Department of Automotive and IT Convergence, Kookmin University, Seoul 02707, Republic of Korea

**Keywords:** 3D segmentation, deep learning, LiDAR sensor, object detection, data annotation, intensity rendering

## Abstract

In the context of LiDAR sensor-based autonomous vehicles, segmentation networks play a crucial role in accurately identifying and classifying objects. However, discrepancies between the types of LiDAR sensors used for training the network and those deployed in real-world driving environments can lead to performance degradation due to differences in the input tensor attributes, such as x, y, and z coordinates, and intensity. To address this issue, we propose novel intensity rendering and data interpolation techniques. Our study evaluates the effectiveness of these methods by applying them to object tracking in real-world scenarios. The proposed solutions aim to harmonize the differences between sensor data, thereby enhancing the performance and reliability of deep learning networks for autonomous vehicle perception systems. Additionally, our algorithms prevent performance degradation, even when different types of sensors are used for the training data and real-world applications. This approach allows for the use of publicly available open datasets without the need to spend extensive time on dataset construction and annotation using the actual sensors deployed, thus significantly saving time and resources. When applying the proposed methods, we observed an approximate 20% improvement in mIoU performance compared to scenarios without these enhancements.

## 1. Introduction

Autonomous vehicles are designed to navigate to programmed destinations without human input, utilizing an array of sensors such as Light Detection and Ranging (LIDAR), Radio Detection and Ranging (RADAR), and vision-based sensors. These sensors act as the vehicle’s eyes, gathering essential information about its surroundings [1,2,3].

Sensor Characteristics in Autonomous Vehicles: Each type of sensor employed in autonomous vehicles has distinct attributes. Vision sensors are adept at recognizing objects and are especially useful in identifying lanes, traffic lights, and traffic signs. Their drawback, however, is their reduced effectiveness in low-light or cloudy conditions, and their inability to measure distances accurately. These issues are typically addressed through sensor fusion with other sensor types [4,5,6].

Distance Measurement and Sensor Fusion: RADAR and LIDAR are superior to vision sensors in terms of distance measurement, using radio waves and laser pulses, respectively, to ensure reliable performance in dark environments. RADAR is particularly useful for long-range measurements critical to adaptive cruise control (ACC) and automatic emergency braking (AEB) systems, due to its capability to detect objects in longitudinal directions despite significant lateral measurement errors. LIDAR is highly valued for its precision in both longitudinal and lateral measurements and is crucial in applications requiring detailed spatial mapping, although it struggles in adverse weather conditions such as rain or snow, where its laser pulses cannot penetrate large particles.

Ultrasonic and Single Sensor Studies: Ultrasonic sensors are primarily utilized for parking assistance due to their high accuracy and dependable performance in poor weather conditions, though they are limited to short detection ranges. Recent research has focused on using single LIDAR sensors for object detection and tracking, leveraging deep learning to accurately classify and measure distances without the need for sensor fusion [7,8].

Deep Learning in Object Detection: Deep learning-based systems are deployed to detect various on-road objects. The effectiveness of these systems hinges on training with accurately labeled datasets, which significantly influences detection accuracy. To address the variability in dataset quality, open datasets of high quality are provided to the research community to facilitate advancements in object detection and to mitigate the limitations associated with dataset construction.

This paper introduces innovative methods aimed at enhancing object detection and tracking using a single LIDAR sensor. The approach involves object segmentation and clustering, as well as tracking to perform object detection:

1. Improvement of Deep Learning Networks: The mIoU (mean Intersection over Union) of object segmentation has been improved by refining network layers. Rigorous training and evaluation helped identify and implement the most effective network configuration.
2. Intensity Rendering and Data Interpolation: We propose a technique to bridge the gap between test data and training data collected from different sensors, thus minimizing physical discrepancies and preventing degradation in detection accuracy.

The proposed system’s efficacy was evaluated in real road environments using an interacting multiple model tracking algorithm that adapts to the dynamic nature of road objects, allowing for a robust object detection and tracking system that operates independently of the sensor diversity in the training dataset.

## 2. Related Research

Traditional segmentation methods and deep learning based segmentation networks are frequently employed for object classification using single LIDAR sensors.

1. Geometrical Characterization Method: This method classifies objects based on their geometrical characteristics in three-dimensional (3D) data, such as the position and number of points in a cluster. This 3D data clustering technique identifies objects when specific conditions, like the size, shape, or intensity of the LIDAR data, are met. Since users define these conditions themselves, this approach can achieve high accuracy. Nonetheless, the results may vary depending on the object’s pose, position, and shape, making traditional classification methods susceptible to environmental changes and necessitating highly complex conditions [9,10].
2. Deep Learning-Based Segmentation Method: This approach utilizes deep learning to segment objects. Data are inputted into a computer, which is then trained to discern rules. Various deep learning methods have been developed to ensure accurate object segmentation despite environmental variations. The fundamental deep learning process consists of constructing multiple deep layers to create a neural network, identifying optimal parameters for each layer, and deriving the most suitable prediction output [11]. Deep learning algorithms are extensively used and have shown promising results in fields such as image, voice, and handwriting recognition.

Deep Learning for 3D Point Clouds: The classification and segmentation of 3D point clouds using deep learning can be categorized into two approaches:

The first uses the entire 3D point cloud as input. The second converts the 3D data into a 2D image for processing. Networks such as PointNet [12], VoxelNet [13], and SqueezeSeg [14,15] illustrate these methods, each with its strengths and limitations in terms of processing speed and accuracy. RangeNet++ is noted for its superior accuracy in converting 3D LIDAR data into 2D images via spherical projection, achieving a high level of segmentation accuracy after filtering [16].

Performance Comparison: The networks like PointNet, PointNet++, SqueezeSeg, SqueezeSegV2, RangeNet21, 53++, etc., are compared in terms of their object recognition accuracy and scanning speeds. SqueezeSegV2, which has the fastest real-time performance, is preferred even if its accuracy is slightly lower, because object detection performance can be adjusted through post-processing, such as clustering.

As shown in Table 1, the results of recent studies using the SemanticKITTI dataset are presented as IoU(%) values for each detectable category in road scenarios, with the far right column indicating the operating speed (ms).

In this paper, we utilize SqueezeSegV2, an evolution of the original SqueezeSeg model, for object segmentation. SqueezeSegV2 retains the general framework of its predecessor but incorporates enhancements such as an adjusted loss function and improved batch normalization techniques. These modifications result in higher segmentation IoU during training. Although the real-time performance remains unchanged, there is a noticeable improvement in segmentation, as evidenced by higher average IoU values for classes such as cars, pedestrians, and cyclists.

To ensure safe driving for autonomous vehicles, especially in high-speed environments, the perception system must operate effectively in real-time and robustly adapt to various road conditions. SqueezeSegV2 was chosen because it offers the fastest processing speed among networks that use intensity values as an input tensor, ensuring that the perception system functions reliably in diverse driving environments.

A comprehensive training dataset is crucial for effective object segmentation using deep learning [22]. Open datasets like the Karlsruhe Institute of Technology and Toyota Technological Institute (KITTI) are invaluable; however, mismatches in data characteristics between the actual sensors used and those in the datasets can lead to errors when the data is applied in real-world algorithms.

LIDAR point cloud data extensively utilize intensity values, which vary significantly across different environmental elements like asphalt, painted lanes, vehicles, and sidewalks. These intensity differences are crucial for distinguishing between various objects based on size and shape within the 3D point cloud data.

According to the research results from experiments on 3D object detection using different LiDAR sensors, a significant difference in accuracy can be observed when the LiDAR sensor used to build the train dataset is different from the one used during testing. Table 2 shows only a subset of the LiDAR-CS benchmark data, where VLD denotes Velodyne and the number following it indicates the number of channels of the sensor. The AP results for each method significantly decrease due to differences in sensors [23,24].

Deep learning models require large datasets to accurately detect objects. If no suitable open dataset exists that uses the same LIDAR sensor, one must undertake the arduous task of logging and labeling data manually. To circumvent this, some studies use game data from simulations like Grand Theft Auto (GTA), which, while readily available, often fails to accurately represent real-world intensity values. Recent methods address this by applying virtual noise to simulator data, rendering the intensity values more akin to those found in real environments, thereby expanding the usable dataset size [25,26].

The LiDAR point cloud generation method in GTA V typically employs a raycasting approach, a function available within the game, which simulates a virtual LIDAR to gather data. GTA V is particularly useful because it automatically annotates each point with an instance level for object classification (e.g., vehicles, pedestrians, cyclists). While this automated method eases the burden of manual annotation, it is not without its flaws—pedestrian data represented as cylinders can lead to significant errors. The Precise Synthetic Image and LiDAR (PreSIL) dataset, introduced by Hurl et al. [27], addresses these inaccuracies by offering more realistic data through improved instance segmentation.

Similarly, the CARLA simulator synthesizes 3D point cloud data using a raycasting method. According to Wang et al. [28], this technique allows for the automatic collection of data, mirroring the KITTI dataset in requiring approximately 15 h for about 1700 frames.

Despite the utility of these synthesized datasets in training, they are not suitable for real-time data evaluation due to physical discrepancies caused by sensor differences, leading to inaccurate results. This paper proposes novel methods of intensity rendering and 3D data interpolation to bridge the gap between training and real data without compromising real-time performance. Our principal contribution is the development of an optimal intensity rendering function that facilitates point-to-point matching across different sensors and improves detection accuracy through data interpolation.

The enhancements in data matching not only elevate the classification accuracy but also enhance the subsequent tracking of classified objects, confirming the effectiveness of our proposed methods. By implementing these algorithms, users can significantly reduce the need for extensive data collection tailored specifically for deep learning applications.

## 3. System Overview

**Figure 1:**
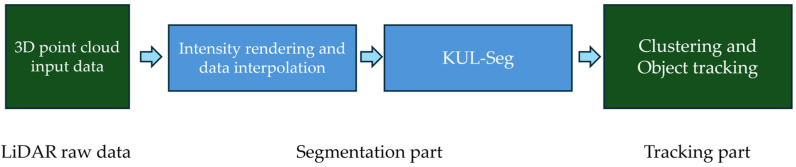
Proposed System Flow Diagram: The system is divided into segmentation and tracking parts. The segmentation part, which is the main contribution of this study, is indicated with blue boxes.

In this research, we developed a perception system utilizing a single 3D LIDAR sensor. As depicted in Figure 1, the system primarily consists of detection and tracking components. The detection segment uses 3D data as input to perform object classification and segmentation through deep learning networks, identifying the targets for tracking. The tracking component incorporates the Interacting Multiple Model Unscented Kalman Filter Joint Probabilistic Data Association (IMM-UKF-JPDA) algorithm, which effectively tracks multiple models, accounting for road conditions.

## 4. KUL-Seg

**Figure 2:**
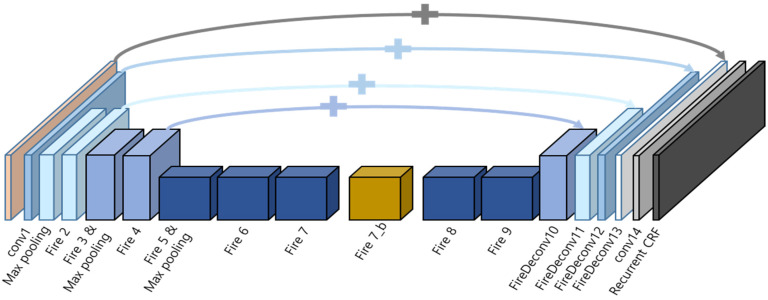
The KUL-Seg network composition with one additional Fire module added. The added Fire module is represented by a yellow box in the diagram.

Figure 2 illustrates the layer structure of the proposed segmentation network, KUL-Seg. While the overall layer composition is similar to the framework of SqueezeSegV2, a new Fire module (Fire 7_b) has been added between Fire modules 7 and 8 to enhance segmentation mIoU. Various attempts were made to configure the optimal network, such as changing the convolution filter size or adding new convolution layers. The KUL-Seg network, proposed herein, demonstrated the most stable results across various tests.

Table 3 presents the performance of networks trained on 10,848 KITTI datasets. A single KITTI dataset contains scene data with a size of 64 × 512 and consists of six tensors: x, y, z, intensity, range and label. The data is provided in numpy format. The configuration utilizing a single fire module 7b yielded the highest accuracy and mean IoU. The IoU for each category of Car, Cyclist, and Pedestrian is presented according to the configuration of the network layer. Each network variant is labeled to indicate modifications: (S) indicates a layer with a smaller filter than the original; (L) indicates a layer with a larger filter; ‘dual pipe’ describes a network configuration where identical convolution layers to the original SqueezeSegV2 are set in parallel prior to deconvolution; (E) represents an expanded network with more fire modules; (R) denotes a network with a reduced number of fire modules, with the specific number of reductions shown in parentheses; ‘Single #b’ refers to a network where an additional fire module is inserted as a single layer; ‘odd’ includes networks with all odd-numbered fire modules added; and ‘second half’ denotes networks where a fire module is added in the latter half. ‘NP’ stands for the original layers without any additional pooling.

To adapt SqueezeSeg for training with 3D data, a conversion to 2D is necessary. This involves automatically extracting a 90° Region of Interest (ROI) from the 3D LIDAR data and performing spherical projection to generate an input image. Each point within the point cloud is characterized by zenith and azimuth angles, calculated as shown in Equations (1) and (2). The data, existing within a 3D space, is projected forward to form the input image. The resultant input tensor is composed of five channels: x, y, z, intensity, and range, and is sampled to a resolution of 64 pixels × 512 pixels. The range value is computed using the Euclidean distance from the origin, based on the LIDAR data’s x, y, and z coordinates.

The azimuth and zenith angles are defined by Equations (1) and (2) as follows:

(1) $$\theta =arcsin\frac{z}{\sqrt{x^{2}+y^{2}+z^{2}}}$$

(2) $$\phi =arcsin\frac{y}{\sqrt{x^{2}+y^{2}}}$$

Considering the forward direction of the 3D point cloud data aligns with the X-axis, the point cloud is projected onto the Y-Z plane using a 45° angle on either side, forming a frontal view. The field of view (FOV) used for capturing the data span of 90°, with the LIDAR data from this region divided into 512 columns (pixels) and each channel data forming 64 rows (pixels), which are then spherically projected onto the Y-Z plane to serve as the network’s input.

SqueezeSeg is a variant of SqueezeNet, characterized by a similar network architecture but differentiated primarily by the dimensions of the input tensors [29]. Unlike SqueezeNet, which utilizes a square input tensor, SqueezeSeg uses a horizontally elongated rectangular tensor measuring 64 pixels by 512 pixels. Consequently, while SqueezeNet downsamples both the height and width during convolution operations, SqueezeSeg only downsamples the width.

**Figure 3:**
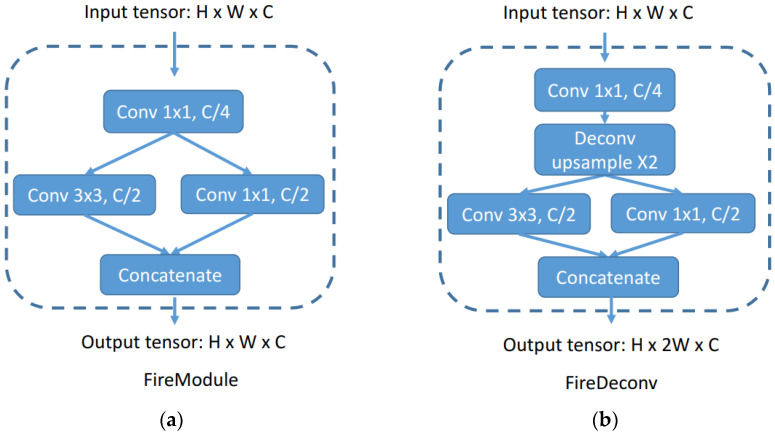
(**a**) represents the layer configuration of the Fire module, while (**b**) represents the layer configuration of the Fire Deconv.

A notable feature of SqueezeSeg’s architecture is its adoption of the shortcut concept, akin to that used in ResNet. This is implemented to mitigate data loss during the downsampling process by relaying an upsampled feature map through a skip connection, as depicted in Figure 3. The convolution layers in SqueezeSeg incorporate the Fire module and Fire Deconv concepts. These are designed to reduce the number of parameters and the computational load typically associated with conventional convolution and deconvolution layers in CNNs.

The Fire module includes a squeeze layer, which uses 1 × 1 convolutions to reduce the channel size. The expand layer, comprised of both 1 × 1 and 3 × 3 filters, performs convolution operations and then concatenates the outputs of the previous layers to restore the channel count while still reducing the overall computational demand.

For instance, if an input tensor’s dimensions are H × W × C and a 3 × 3 convolution filter is employed, the number of parameters would traditionally be calculated as $9C^{2}$ and the computation as $9{HWC}^{2}$. In contrast, within the Fire module, the number of parameters is reduced to $\frac{3}{2}C^{2}$, and the computation to $\frac{3}{2}{HWC}^{2}$. Thus, the Fire module significantly decreases both the number of parameters and the computational requirements.

The reduction in computational time, achieved through the use of fewer parameters, ensures performance akin to that of AlexNet. However, the use of a SqueezeSeg network is particularly beneficial in applications like autonomous vehicles, where real-time performance is critical.

As the image passes through each layer, the convolution filters detect the feature points of the object and determine the class to which it belongs. In this study, object classification was segmented into four categories: vehicle, cyclist, pedestrian, and unknown. The model was trained using a dataset comprising 10,848 entries from the KITTI database, enabling it to effectively learn and classify diverse road objects.

## 5. Intensity Rendering and 3D Points Interpolation

In this study, the KITTI dataset, utilized for training, was collected using a Velodyne 64-channel LIDAR sensor, model HDL-64E (Velodyne, San Jose, CA, USA). When this dataset is applied to different LIDAR sensor data, such as those from a sufficiently trained SqueezeSeg network, there is a notable reduction in training effectiveness. This reduction is attributed to the different resolutions and intensity specifications of each sensor, as the SqueezeSeg network relies on five specific tensors for features: x, y, z, intensity, and range. Consequently, discrepancies between training and testing sensors can significantly impact the accuracy of the SqueezeSeg network. Although the KITTI dataset is widely used, the actual input sensor data often differ from the training dataset, which poses a challenge for model performance.

The original Velodyne HDL-64E sensor, integral to the KITTI dataset compilation, features a 360° horizontal field of view, a horizontal angular resolution of 0.08°, a vertical FOV of 26.9°, and a vertical angular resolution of 0.4°. In contrast, the Ouster OS1-64 LIDAR sensor (Ouster, San Francisco, CA, USA) used in our experiments possesses a 360° horizontal FOV, a horizontal angular resolution of 0.35°, a vertical FOV of 45°, and a vertical angular resolution of 0.35°. The differing resolutions between these two sensors result in a gap in the data when projected onto a 2D plane of 64 × 512 pixels for the SqueezeSeg network. To address this issue, we employed linear interpolation to adjust for the resolution differences, setting the data quantity equally.

**Figure 4:**
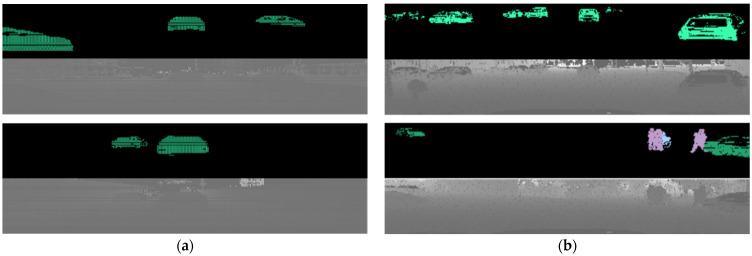
(**a**) Projected image using raw data of Ouster LIDAR with a blank pixel caused by sensor resolution; (**b**) Projected image with linear interpolation for covering the blank pixel. In (**a**), the space between pixels is empty in the horizontal direction due to the difference in resolution, but (**b**) consists of continuous data without spaces.

Linear interpolation effectively reduces the gap in the horizontal angular resolution between the two sensors. As demonstrated in Figure 4, the interpolated data show a significant improvement. Figure 4a displays the initial projected image from the raw data of the Ouster sensor, where some rows and columns appear empty due to poor resolution. In contrast, Figure 4b shows the image post-interpolation, where the gaps are filled, providing a complete and improved resolution image.

This linear interpolation process is crucial for mitigating differences in sensor resolution. While it can increase the data volume by approximately a factor of eight in some cases, which might potentially affect real-time performance due to higher computational demands, this impact is minimized as only the frontal data are utilized in this context. This approach allows for the more accurate application of models trained on the KITTI dataset to different sensor types, ensuring a more robust and reliable performance in varied operational environments.

**Figure 5:**
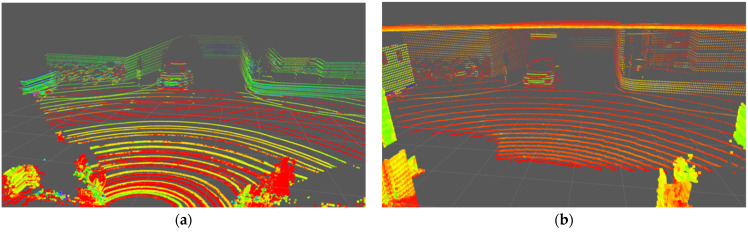
Sensor intensity and resolution comparison in same environment. (**a**) Point cloud data of Velodyne HDL-64e; (**b**) Point cloud data of Ouster OS1-64. The color of each point is displayed in a different color according to the intensity value distribution of each sensor.

In this study, differences in the intensity values between sensors affected the outcomes of the SqueezeSeg model, as illustrated in Figure 5. For example, the Velodyne sensor has intensity values ranging from 0–255, while the Ouster sensor records values approximately between 0–9000. An identical point on the floor, when scanned by both sensors at the same time and location, returned intensity values of 5 for Velodyne and 113 for Ouster. The intensity among the input tensors of the five channels is utilized as a critical feature to distinguish objects in the LIDAR point cloud, akin to how color information is used in vision sensor images.

To evaluate the correlation between the Velodyne HDL-64E and the Ouster OS1-64, data were compared after scanning the same environment simultaneously from the same position, and the intensity correlation was quantitatively analyzed. The decision against normalizing intensity data was due to the substantial scale differences between the two sensors, which, when normalized, led to greater errors in intensity values.

**Figure 6:**
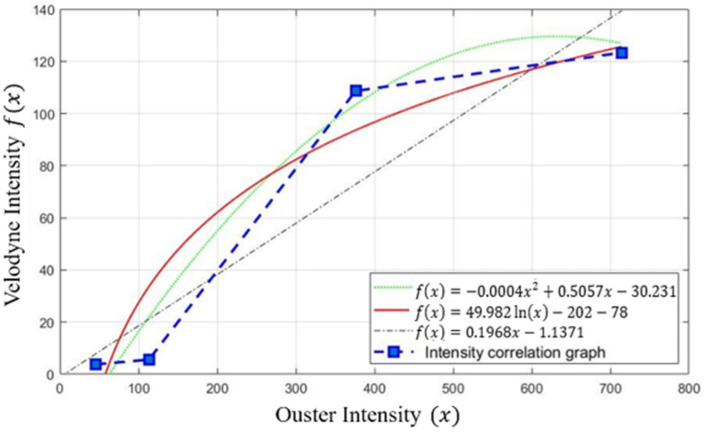
The graph of intensity correlation between sensors using different approximation functions. The green line indicates the polynomial function, the red line indicates the logarithmic function, and the black dotted line indicates the linear function.

The persisting differences were further confirmed, as depicted in Figure 6 and Table 4, by analyzing approximately 3000 points from frequently detected parts. We applied the least squares method to approximate a relationship between the two sets of sensor data. While intensity data can vary by situation, it appeared visually similar to Velodyne data when compared with non-rendered data. Moreover, the evaluation accuracy of the SqueezeSeg outputs was higher when using these processed data.

**Figure 7:**
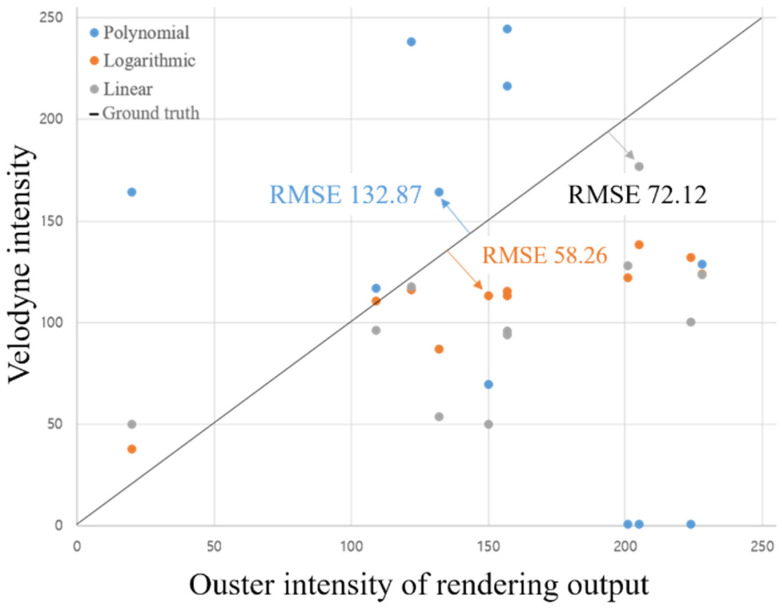
The graph of the root mean square error of each function. The blue point is a polynomial function, the orange point is a log function, and the gray point is a linear function, and the black line crossing the graph represents the ground truth.

For clarity, each part, such as different vehicle sections like the bonnet, radiator grille, and tires, was represented by average intensity values. Graphs representing these averages were categorized into four groups: vehicle, truck, ground, and pillar. Figure 7 presents a graph that compares point-to-point matches across all categories to identify the most suitable function from the approximate equations provided in Figure 6. We computed the root mean square error (RMSE) for each function using the Ouster data and the ground truth data from Velodyne LIDAR. Among various tested functions, the logarithmic function consistently showed the lowest RMSE values, indicating the closest match to the actual data. This finding led to the adoption of the logarithmic function as the primary rendering function, as demonstrated in Figure 7 and detailed in Table 5.

**Figure 8:**
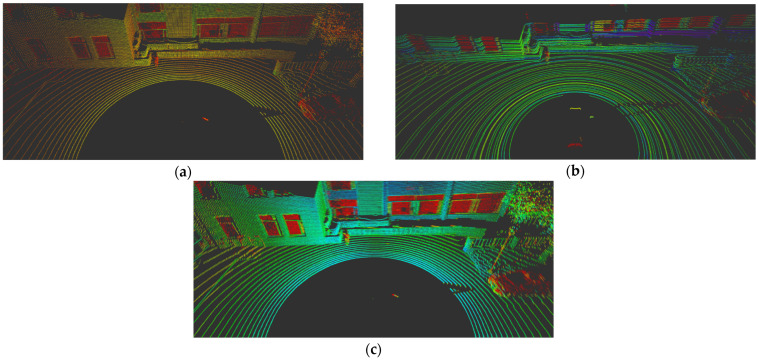
Intensity rendering comparison results. (**a**) Ouster raw data, (**b**) Velodyne raw data, (**c**) Intensity rendering output of Ouster data with logarithm function. If you look at the figure above, which expresses the intensity value in color, you can intuitively see that the value of (**c**) is much closer to the value of the comparison group (**b**) than (**a**).

**Figure 9:**
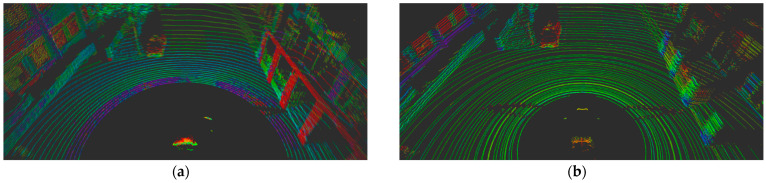

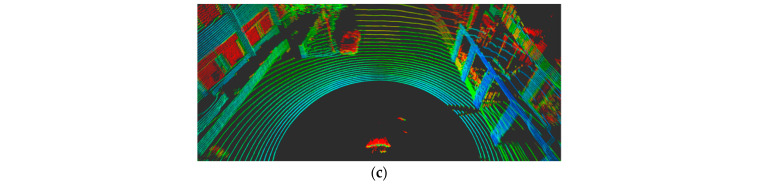
Results of intensity rendering with different rendering functions. (**a**) Intensity rendering output with 2nd order polynomial function, (**b**) Velodyne raw data, (**c**) Intensity rendering output with logarithmic function. As with the RMSE value comparison result in Figure 7, it can be seen that (**c**) is most similar to the Velodyne data when intensity data is expressed by color.

The colors represented in each image of Figure 8 are normalized according to the rainbow color spectrum, ranging from red to purple, based on the intensity distribution of the LiDAR sensor. As shown in the images, the application of the logarithmic function results in a color distribution for the Velodyne LiDAR data and Intensity that appears similar. Figure 9 displays the rendering results for each function, and the detection performance was compared using IoU. The logarithmic function not only yielded the highest accuracy but also demonstrated superior performance in the mAP results, as outlined in Table 5. This confirms the efficacy of using a logarithmic rendering function for adjusting intensity values between different LIDAR sensors, enhancing the overall accuracy of the SqueezeSeg model.

## 6. Object Tracking

Euclidean clustering was utilized to cluster the segmented objects, with Random Sample Consensus (RANSAC) serving as the ground removal algorithm. Using RANSAC to eliminate ground points, data inaccurately labeled as vehicles were removed, enhancing the accuracy of object tracking [30]. It is common in such cases for adjacent points, like ground points or other objects, to be mistakenly grouped together. The labels for each point were assigned by the SqueezeSeg network. Only clusters where the share of the object label exceeded 70% were designated as target clusters for tracking. In our experiments, the false positive ratio of the SqueezeSeg output labels in each segment did not exceed 20%, while the true positive ratio remained above 80%. Setting the threshold at 70% was crucial, as the noise generated during the recovery of the 3D image from the 2D image could misrepresent distant objects as being closer in the 2D image. To mitigate this, clusters with low classification label occupancy were excluded from tracking. The tracker then followed the objects that had been filtered through Euclidean clustering, free from noise. Each label contains the object’s ID, relative speed, and distance from the center point of the tracked object; the object type is visualized by color.

For multi-object tracking, the Interacting Multiple Model Unscented Kalman Filter Joint Probabilistic Data Association algorithm was employed [31]. The different kinetic properties of vehicles, pedestrians, and cyclists necessitated the application of distinct parameters in the tracking. The dynamic models in the IMM filter, such as Constant Velocity (CV), Constant Turn Rate Velocity (CTRV), and Random Motion (RM), were configured based on the tracked object’s behavior.

IMM is an algorithm that utilizes N parallel filter banks and is commonly employed alongside JPDA in complex tracking systems like missile defense and aircraft tracking [32]. The core components of an IMM algorithm include interaction, filter bank, model probability update, and measurement fusion. Mode transition probabilities follow a Markov chain, and the initial probabilities can be set by the user and further refined by Monte Carlo simulation outcomes. Without these probabilities, the IMM operation would resemble a static MM algorithm.

The IMM algorithm operates by determining the next mode from a mixture of all previous filter state estimates. Each mode’s filtering steps are executed in parallel within the filter bank, encompassing prediction and update processes. The updated state estimates are subsequently combined across all filters, and parameters such as mixed mode, state, and covariance are used to compute the probability of the next mode [33].

The UKF model within the filter bank excels in handling complex nonlinear models better than the Extended Kalman Filter. This superiority is due to the sigma sample points being processed through a nonlinear function, resulting in more accurate mean and covariance estimates than those provided by the EKF, which simplifies the nonlinear model through linearization.

**Figure 10:**
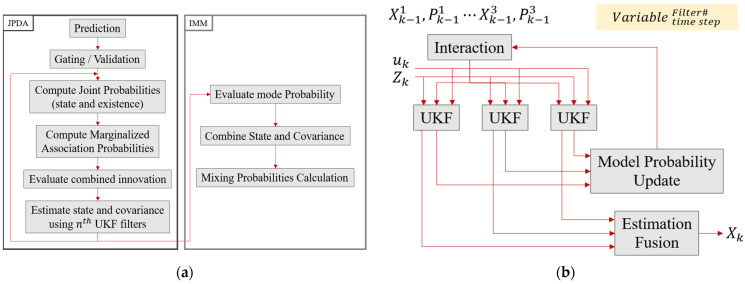
The flow chart of IMM-UKF-JPDA. The contents of each stage of JPDA and IMM are expressed in the box flow chart above, and in the graph below, the overall algorithm flow is expressed as a graph.

Figure 10 illustrates the detailed sequence of the tracking modules, represented by a flowchart (a) and a diagram of the tracking module process (b). The IMM-UKF-JPDA comprises four stages: interaction; prediction and measurement data association; mode probability update; and combination. The process starts with IMM probability mixing in the interaction stage, where mode transition probabilities are calculated. Next, in the UKF, sigma points are selected, propagated through the transfer function, and used to predict measurement and covariance. The state measurement cross-covariance matrix and UKF Kalman gain are then computed. In the data association stage, similar to traditional PDA filters, the updated state and covariance are determined by the association probabilities. Finally, the mode probabilities are updated, and outputs from each filter are combined to produce the final state and covariance. The variables used in the flowchart include state X, covariance P, control vector u, and measurement Z, with superscripts and subscripts denoting the number of filters and time steps, respectively [34].

## 7. Experiments

The PC utilized in the experiment was configured with ROS Kinetic 1.12.17, Python 2.7, and TensorFlow 1.4, running on Ubuntu 16.04 LTS. The network training was executed using an Nvidia GTX 1080 Ti GPU (Nvidia, Santa Clara, CA, USA), and field tests were conducted on various roads with a LIDAR system mounted on mid-sized SUVs. SqueezeSegV2, developed in Python with tensorflow-gpu 1.4, processes the detected points. Subsequently, C++-based Euclidean clustering and object tracking classify the data. The data transmission and reception between nodes were facilitated using ROS Topic.

To assess the performance of the network, an evaluation test was carried out on both the original and intensity-rendered Ouster data using the SqueezeSegV2 network, which had been trained on the KITTI dataset. The test dataset included ‘Car’, ‘Cyclist’, and ‘Pedestrian’, detected by the Ouster sensor, appearing 3421, 17, and 13 times, respectively, across 867 frames collected from highways, city roads, alleys, and residential areas. As indicated in Table 6, there was a noticeable improvement in the accuracy of identifying the vehicle, cyclist, and pedestrian categories. When compared to the values in the “No rendering & interpolation” row, which represent the results using the existing segmentation network, it can be observed that applying the logarithmic function rendering and 3D point cloud data interpolation methods proposed in this study improves the IoU values in all areas. Specifically, Car improved by approximately 7%, Cyclist by about 28%, and Pedestrian by around 43%.

**Figure 11:**
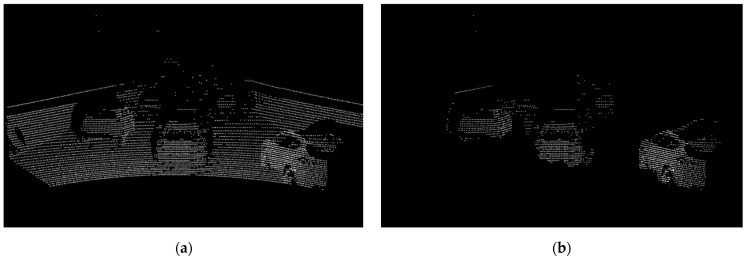
The image of SqueezeSegV2 output. (**a**) Rendered and interpolated point cloud data, (**b**) result of SqueezeSegV2. Object detection was performed with SqueezeSegV2 using the preprocessed value (**a**) to obtain the result, (**b**). By using the data in (**b**), the model goes through the clustering process and removes non-vehicle guard rails and floor surfaces that have not been removed.

During real road tests, as depicted in Figure 11, it is evident that vehicle data are marked in white. For semantic segmentation, the mean Intersection over Union metric is used, defined by Equation (3), where TP, FP, and FN represent true positives, false positives, and false negatives, respectively. The variable “c” denotes the class, while uppercase “C” represents the number of classes:

(3) $$mIoU=\frac{1}{C}\sum_{c=1}^{C}\frac{TP_{c}}{TP_{c}+FP_{c}+FN_{c}}$$

The differences in angular resolution and intensity range between Velodyne’s HDL-64 LIDAR, used in training, and Ouster’s OS1, used in experiments, presented challenges in object classification. Nevertheless, the adaptation of sensor characteristics through intensity rendering resulted in highly accurate classification, particularly because the same optimally set hyperparameters were applicable. Notably, smaller objects like cyclists and pedestrians, which typically exhibit fewer features than vehicles, demonstrated significant improvements in accuracy post-intensity rendering.

**Figure 12:**
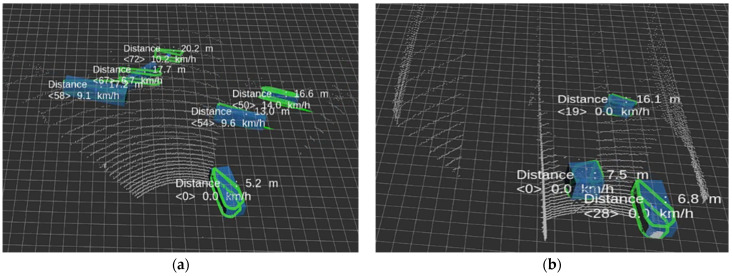
The image tracking results. (**a**) Urban environment; (**b**) highway environment. Among the tracking results, the distance to the center point of the object, target ID, and relative speed were visualized.

Field experiments were conducted at an urban road intersection and on a highway, under clear weather conditions to avoid complications from rain or snow. As illustrated in Figure 12, at the urban intersection, vehicles turning or driving straight at a five-way signal-controlled junction were detected and tracked. On the highway, nearby moving vehicles were tracked as the test vehicle drove straight.

The experimental setups compared the performance of data with and without the applied intensity rendering and data interpolation, using the same tracking algorithms. The results of these comparisons are documented in Table 7, demonstrating the effectiveness of the proposed adjustments in real-world conditions.

During the evaluation, tracking tests were performed on two distinct datasets, consisting of 289 and 365 frames, respectively. In both instances, the data processed by the proposed algorithm demonstrated considerably more stable outcomes across various scenarios. These metrics included false alarms—where non-vehicles were incorrectly identified as vehicles, missed detections—where vehicles were not identified, and ID switch counts—where the shape of a detected object changed frequently enough to necessitate a change in the tracking ID.

Due to differences in 3D point data distribution, a single object can be clustered as two separate objects in the segmentation results. However, when applying the method proposed in this study, the segmentation results improve, preventing such issues. Consequently, this leads to enhanced tracking stability. As evidenced, when the output data from the deep learning network become unstable, it adversely affects the tracking algorithm’s performance. This underscores the importance of stable and reliable object detection data as a prerequisite for efficient tracking performance. The proposed algorithm’s ability to maintain stability across different test conditions highlights its robustness and potential applicability in real-world autonomous driving systems [33].

## 8. Conclusions

In this study, we provided a rendered 3D LIDAR point cloud as the input to a SqueezeSeg network, which was trained using the KITTI dataset. To enhance object classification, we removed the ground from the KITTI data for better clustering. The differences between the training data and the test data significantly impact classification accuracy; therefore, we corrected the Ouster data, used as test data, by mapping the intensity relationships and matching the resolution differences to conditions similar to the KITTI data using linear interpolation. This process led to an improved detection performance, even with different sensors. By implementing our proposed solution and integrating various sensors, we effectively compensated for sensor variations and prevented degradation in the detection accuracy of the deep learning network.

The quantity of high-quality training data is directly related to the accuracy of the network. Given the challenges in collecting such data, our rendering techniques offer solutions across multiple areas.

In this study, the proposed method improved the segmentation IoU for Car, Cyclist, and Pedestrian by approximately 7%, 28%, and 43%, respectively, compared to the existing methods, achieving a 20% improvement in mIoU. When applying this method to object tracking across two datasets in different environments, the number of tracking failures, which is the sum of false alarms, missed detections, and ID switches, was reduced to about 18% and 8%, respectively, compared to when the method was not applied.

Furthermore, the reliability of classifications using deep learning varies depending on the road environment. To address this, we combined deep learning with the Euclidean clustering method, where an object is recognized as a tracking target only if the classified points in the cluster exceed a certain threshold. This approach helps remove static obstacles, such as trees or walls.

To validate the proposed method, tests were conducted in static and various dynamic environments, including downtown areas, highways, and suburban roads. Testing in only static conditions does not account for factors like vehicle vibration due to road irregularities and data processing time delays related to vehicle speed, leading to significant differences in results. However, after extensive testing in actual road environments, the results confirmed that the impact of environmental changes is minimal.

This study primarily focused on reducing the differences between training and test datasets by comparing Velodyne’s HDL-64 and Ouster’s OS1 LIDAR. Future work will explore comparisons with other sensors and datasets to further validate and expand the applicability of our methods.

## Figures and Tables

**Table 1:** Semantic segmentation result on Semantic KITTI.

Method	Road	Car	Truck	Bicycle	Motor Cycle	Other Vehicle	Person	Bicyclist	Motor Cyclist	Speed (ms)
SqueezeSegV2 [15]	88.6	81.8	13.4	18.5	17.9	14	20.1	25.1	3.9	20
RangeNet53++ [16]	91.8	91.4	25.7	25.7	34.4	23	38.3	38.8	4.85	83.3
SqueezeSegV3 [17]	91.7	92.5	29.6	38.7	36.5	33	45.6	46.2	20.1	238
PointNet++ [18]	72	53.7	0.9	1.9	0.2	0.2	0.9	1	0	5900
TangentConv [19]	83.9	90.8	15.2	2.7	16.5	12.1	23	28.4	8.1	3000
RandLA-Net [20]	90.5	94.2	43.9	29.8	32.2	39.1	48.4	47.4	9.4	880
JS3C-Net [21]	88.9	95.8	54.3	59.3	52.9	46	69.5	65.4	39.9	471
SPVNAS	90.2	97.2	56.6	50.6	50.4	58	67.4	67.1	50.3	259
Cylinder3D	92.2	97.1	50.8	67.6	63.8	58.5	73.7	69.2	48	131
RPVNET	93.4	97.6	44.2	68.4	68.7	61.1	75.9	74.4	43.4	168

**Table 2:** Cross evaluation on LiDAR-CS benchmark result.

	Val	VLD-64				VLD-32				VLD-16			
Train													
	Methods	mAP	Car	Truck	Ped.	mAP	Car	Truck	Ped.	mAP	Car	Truck	Ped.
VLD-64	PointPillar	64.16	82.19	88.04	31.76	36.29	51.50	57.68	19.45	26.42	40.96	41.85	14.82
	SECOND	67.30	82.09	87.85	31.28	34.87	45.77	50.89	19.42	22.99	35.16	32.96	12.56
	POINTRCNN	41.69	57.89	63.80	13.69	27.56	35.26	38.80	12.51	21.72	28.87	27.86	9.70
	PV-RCNN	71.97	89.15	90.95	30.74	39.94	54.38	57.49	17.51	27.47	42.16	37.60	11.29
	CenterPoint	78.00	86.08	88.18	59.23	41.86	48.51	53.72	32.92	27.70	36.34	35.25	20.66
VLD-32	PointPillar	42.96	60.76	71.64	19.94	46.08	64.11	75.60	23.64	36.55	52.73	55.40	21.75
	SECOND	41.28	56.69	63.00	20.76	48.40	62.01	73.77	26.45	37.89	50.44	54.04	22.08
	POINTRCNN	36.11	55.51	61.29	9.13	35.76	49.09	51.23	16.11	33.47	43.95	44.32	16.27
	PV-RCNN	44.97	66.61	63.68	18.38	53.45	71.48	79.37	24.64	43.61	59.75	61.38	21.54
	CenterPoint	51.23	59.74	67.01	34.52	56.94	63.55	73.26	41.50	43.83	51.56	52.35	34.28
VLD-16	PointPillar	25.50	35.13	46.32	13.84	35.83	53.70	54.45	19.27	39.70	57.09	64.36	22.64
	SECOND	19.22	25.26	38.50	12.00	39.68	54.74	61.37	20.50	39.07	54.33	61.72	20.26
	POINTRCNN	33.38	48.78	62.37	5.55	38.47	49.09	56.73	17.92	37.45	46.43	49.93	19.61
	PV-RCNN	17.65	26.66	40.64	5.12	46.18	65.20	72.13	21.27	46.28	64.43	69.61	22.63
	CenterPoint	17.42	26.03	34.02	5.39	48.69	56.36	62.15	34.86	47.56	54.49	61.44	34.39

**Table 3:** Network evaluation results.

Category	Dual Pipe	Dual Pipe (S)	Dual Pipe (L)	Dual Pipe (XS)	Dual Pipe (XL)	Dual Pipe (NP)	Dual Pipe (E)	Dual Pipe (R)	Dual Pipe (R3)	Dual Pipe (RNP)	Dual Pipe (R4)
Car	0.78	0.81	0.81	0.80	0.80	0.81	0.80	0.80	0.80	0.79	0.81
Pedestrian	0.49	0.41	0.34	0.41	0.54	0.53	0.47	0.30	0.52	0.23	0.47
Cyclist	0.38	0.53	0.52	0.54	0.39	0.41	0.43	0.54	0.43	0.49	0.46
mIoU	0.55	0.58	0.56	0.58	0.58	0.58	0.57	0.55	0.58	0.50	0.58
Category	Single (S)	Single (Odd)	Single (2nd half)	Single 2b(S)	Single 3b(S)	Single 4b(S)	Single 5b(S)	Single 6b(S)	Single 7b(S)	Single 8b(S)	Single 8b(L)
Car	0.76	0.76	0.77	0.77	0.76	0.76	0.77	0.77	0.76	0.78	0.77
Pedestrian	0.37	0.23	0.23	0.40	0.39	0.45	0.48	0.46	0.24	0.28	0.26
Cyclist	0.38	0.48	0.48	0.38	0.39	0.34	0.37	0.34	0.49	0.56	0.53
mIoU	0.50	0.49	0.49	0.52	0.52	0.52	0.54	0.52	0.50	0.54	0.52
Category	Single all (Same)	Single 2b (Same)	Single 3b (Same)	Single 4b (Same)	Single 5b (Same)	Single 6b (Same)	Single 7b (Same)	Single 8b (Same)			
Car	0.78	0.80	0.80	0.80	0.80	0.79	**0.84**	0.78			
Pedestrian	0.22	0.30	0.30	0.52	0.30	0.51	**0.72**	0.40			
Cyclist	0.48	0.55	0.55	0.39	0.55	0.40	**0.54**	0.41			
mIoU	0.49	0.55	0.55	0.57	0.55	0.57	**0.70**	0.53			

**Table 4:** Intensity value comparison.

Category	O Intensity	V Intensity	Ratio
Vehicle	45.91	3.78	0.08
Truck	714.50	123.38	0.17
Ground	113.28	5.60	0.05
Pillar	376.01	108.64	0.29

**Table 5:** RMSE of each function.

Functions	Linear	2nd Order Polynomial	Logarithm
RMSE	72.12	132.87	58.26

**Table 6:** Table of mIoU performance comparison according to the rendering function.

Intensity Rendering Method	Car	Cyclist	Pedestrian	mIoU
No rendering and interpolation	70.9	26.5	32.7	43.4
Linear function	71.7	22.3	12.5	35.5
Polynomial function	72.2	26.9	20.4	39.9
**Logarithm function**	**75.9**	**33.6**	**46.7**	**52.0**

**Table 7:** Tracking result.

Scene	Frame	Object	False Alarm	Missed Detection	ID SW	Remarks
Dataset 01	289	45	13	117	62	
			3	24	7	Both methods applied
Dataset 02	365	4	72	27	22	
			4	3	3	Both methods applied

## Data Availability

Data are contained within the article.
